# Genome Sequencing and Characterization of *Bacillus velezensis* N23 as Biocontrol Agent against Plant Pathogens

**DOI:** 10.3390/microorganisms12020294

**Published:** 2024-01-30

**Authors:** Panlei Yang, Qingchao Zeng, Wenxiao Jiang, Luotao Wang, Jie Zhang, Zhenshuo Wang, Qi Wang, Yan Li

**Affiliations:** Department of Plant Pathology, College of Plant Protection, China Agricultural University, Beijing 100193, China; yangpanlei1993@163.com (P.Y.); zengqc1992@cau.edu.cn (Q.Z.); jwxiao09@163.com (W.J.); x1970218067@163.com (L.W.); jiezhang207304@163.com (J.Z.); zhenswang@163.com (Z.W.); wangqi@cau.edu.cn (Q.W.)

**Keywords:** *Bacillus velezensis*, biocontrol, lipopeptide, genome mining

## Abstract

The overuse of chemical fungicides against fungal pathogens adversely affects soil and plant health, resulting in environmental problems and food safety. Therefore, biocontrol is considered as an environmentally friendly and cost-effective green technique in environmental protection and agricultural production. We obtained a bacterial strain N23 from a contaminated plate which showed significant inhibition to anthracnose. The strain N23 was identified as *Bacillus velezensis* based on 16S rRNA gene, *gyrA* gene, and whole-genome sequence. The bacterium N23 was able to suppress the mycelial growth of numerous plant pathogenic fungi on solid media. Tomato seeds treated with strain N23 showed significantly higher germination levels than untreated ones. Moreover, strain N23 effectively reduced the lesion area of pepper anthracnose disease in planta. The gene clusters responsible for antifungal metabolites (fengycin, surfactin, and iturin) were identified in the genome sequence of N23 based on genome mining and PCR. Furthermore, methanol extracts of the bacterial culture caused significant inhibition in growth of the fungal *Colletotrichum* sp. and *Botrytis cinerea*. These findings suggested that *B. velezensis* N23 could be a potential biocontrol agent in agricultural production and a source of antimicrobial compounds for further exploitation.

## 1. Introduction

Food production through agriculture is crucial to meet food demand on a global scale. However, the phytopathogen, which affects plant health, poses a chronic threat to food production [1]. The production of food crops is severely reduced every year due to various kinds of plant diseases [2]. Effective measures are necessary to prevent crop loss. Over the past few decades, agrochemicals have become an increasingly relied-on method for addressing and tackling the problem [3]. However, the excessive use of chemical tools to control plant diseases and increase crop yield has led to serious results such as irreversible degradation of soil quality, and drug resistance [4,5]. Owing to the harmful effects of chemicals on the environment, numerous countries have successfully implemented measures to reduce the use of chemical agents [5]. Additionally, they are actively researching and developing eco-friendly alternatives such as green pesticides and biocontrol agents to achieve sustainable disease control and improve crop yield [3,4]. Beneficial bacteria are increasingly being used as an alternative to chemical pesticides in plant protection, leading to a steady growth in plant disease control [6]. Several species of bacteria, like *Pseudomonas*, *Bacillus*, and *Streptomyces*, are predominantly studied and increasingly marketed as biological control agents due to their environmental friendliness and diverse functions including promoting plant growth, controlling diseases, ameliorating soil, and so on [7]. Except their functions of mentioned above, the biocontrol agents could also inhibit the plant pathogen by producing some chemical compounds and active the immune systems of the plant to suppress the phytopathogens in sustainable agriculture. It is worth nothing that the complex environment inevitably affects the survival of the benefit microorganisms and the biocontrol ability [8,9]. Therefore, biological preparations made from spore-forming *Bacillus* sp. are preferred due to their long-term viability, which facilitates the development of commercial products [10]. Some *Bacillus* have increasingly played a key role in many areas, such as biofertilizers, biomaterials, and medicine [11,12]. Notably, it is estimated that biological control agents, based on *Bacillus* sp., make up approximately 50% of commercially available bacterial biocontrol products [13].

The genus *Bacillus*, belonging to the Firmicutes phylum, is a large group of Gram-positive bacteria. In nutrient depletion and kinds of environmental stresses, they are capable of forming outstandingly stable dormant endospores [14,15]. The bacteria from these groups are exceptionally ubiquitous since they can occupy a large variety of ecological niches, such as soil, water, and air, as well as on surfaces and rhizospheres of plants [16]. Species such as *B. subtilis*, *B. velezensis*, and *B. cereus* in the *Bacillus* genus are applied in promoting plant growth and controlling plant diseases [17,18,19]. The mechanisms which the biocontrol strains are mediated can be categorized as direct and indirect. The biocontrol strains, on the one hand, aid in resource acquisition for plants and the modulation of plant hormone levels, and on the other hand, they aim to reduce the negative effects of various pathogens on plant growth and development, including competition for nutrients, niche exclusion, induced systemic resistance, and the production of antibiotic metabolites [7]. It has been reported that controlling pathogens biologically may need a combination of mechanisms, including competition for space or nutrients, the induction of systemic resistance to pathogens, and antibiotic production [20,21,22].

Antibiotic production may play a vital function in their biocontrol activities. *B. velezensis* FZB42 has as much as 8.5% of its genome devoted to the synthesis of antibiotics and siderophores by the non-ribosomal pathway [23]. From the biological control perspective, one of the essential features is their ability to produce structurally diverse Bacillus lipopeptides (LPs) synthesizing by non-ribosomal pathway. These LPs are the major contributors to the biocontrol activity of the Bacillus genus. For instance, fengycin and bacillomycin D, produced by *B. velezensis* FZB42, show synergistic antagonistic activity against the pathogen *Fusarium oxysporum* [24]. For the *Bacillus* strains UMA6614 and UMAF6639, the lipopeptides iturin and fengycin directly mediate the antagonism toward fungal and bacterial pathogens [25,26]. In addition, lipopeptides have also been known to induce host plants’ systemic resistance and facilitate the beneficial bacteria colonization of the plant roots by biofilm formation. For example, the bacillomycin D contributes to biofilm formation in strain *B. velezensis* SQR9 [27]. Surfactin and bacillomycin L contribute differently but synergistically to the biocontrol of rice sheath blight in *B. subtilis* 916 through its antifungal activity, biofilm formation, and colonization [28]. *Colletotrichum* species cause devastating diseases such as anthracnose and fruit rots on a broad range of plant species such as pepper, strawberry, apple, and so on [29,30]. However, there are few biocontrol bacteria to control these pathogens [31]. Therefore, the exploitation of beneficial microorganisms with the potential of biocontrol will be helpful to the development of biocontrol agents.

In this study, we isolated a strain N23 from a soiled plate because of its strong inhibition against plant pathology on the plate. We focused on exploring biocontrol efficient of the strain N23, which could be used to control pepper and strawberry anthracnose caused by *Colletotrichum*. The purpose of this study was to (1) identify strain N23 based on morphological and whole-genome sequence and (2) explore the potential biological control mechanism of *B. velezensis* N23 that inhibited the growth of *Colletotrichum* sp. In the present study, the strain N23 was classified as *B. velezensis* and also showed its potential ability as a biocontrol agent for the management of anthracnose disease. The study provides a foundation for further studies of functions and facilitate genetic engineering of *B. velezensis* N23 to promote agricultural and industrial applications.

## 2. Materials and Methods

### 2.1. Bacterial Strains and Growth Conditions

*B. velezensis* N23 was grown at 37 °C on Luria-Bertani (LB, 10 g tryptone; 5 g yeast extract; and 10 g NaCl; 1000 mL ddH_2_O; pH 7.0; 15 g/L agar powder was added to LB for solid medium). For assays of biofilm formation, we used MSgg liquid medium following the described protocol. The MSgg medium consisted of 100 mM MOPS (morpholine propane sulfonic acid, pH 7.0), 5 mM potassium phosphate (pH 7.0), 2 mM MgCl_2_, 700 μM CaCl_2_, 50 μM MnCl_2_, 50 μM FeCl_3_, 1 μM ZnCl_2_, 2 μM thiamine, 0.5% glycerol, 0.5% glutamate, 0.5% tryptophan, and 0.5% phenylalanine. Then, 15 g/L agar powder was added to MSgg for solid medium [32].

### 2.2. In Vitro Antifungal Activity

The plate confrontation assay was performed as described with some modification [33]. In brief, the pathogenetic fungi, such as *Colleotrichum* sp., *Alternaria solani*, *Fusarium* sp., *Botrytis cinerea*, and *Exserohilum turcicum*, were cultivated on potato dextrose agar medium (PDA, 200 g potato, 20 g glucose, 15 g agar powder, 1000 mL ddH_2_O) at 20 °C or 28 °C for 5–7 days. A pathogen disc with an approximate diameter of 5 mm was then transferred to the center of a PDA plate (90 mm). After 1 day of incubation, 2 µL culture of strain N23 in LB broth for 16 h was spotted on the PDA plate, 2.5 cm away from the pathogen disc. Plates inoculated only with the pathogen were used as the control. After 5–7 days, the antifungal activity was evaluated by measuring and comparing the mycelial radial growth of the pathogen in treatment and control.

### 2.3. Biofilm Formation and Swarming Motility Assay

In brief, 5 mL LB liquid cultures were prepared with shaking (200 rpm) at 37 °C to OD_600_ = 0.8, 1 mL of cells were collected by centrifugation at 6000× *g* for 5 min, washed with phosphate-buffered saline (PBS, 137 mM NaCl, 2.7 mM KCl, 10 mM Na_2_HPO_4_, and 2 mM KH_2_PO_4_), and resuspended in 100 µL PBS. Swarming motility of N23 was tested using standard protocols with minor modification. LB plates containing 0.7% agar were dried in a laminar flow hood for 20 min and then 3 µL of the cell suspension was spotted on the center of each plate [34]. Briefly, 0.7% agar LB plates were dried on the benchtop for 10 min, and then 5 µL of the N23 resuspension was spotted in the center of each plate. LB broth was the control. The plates were at room temperature for 5 h to facilitate the bacteria swarming and growth. To visualize the swarming zone, the plates were dried on the benchtop for 2 h and then incubated at 37 °C for 6 h. The diameter of the swarming zones was measured and recorded.

The pellicle and biofilm formation of strain N23 were monitored in the MSgg medium. Initially, 4 mL of MSgg medium was inoculated in a 12-well plate and then cultured in the 25 °C incubator for 36 h. LB broth was the control. To quantify the pellicle of strain N23, the liquid medium under the pellicle was gently removed and the pellicle was carefully washed three times with 3 mL of sterile saline. It was then fixed with 2 mL of 99% (*v*/*v*) methanol for 15 min and air-dried. The dried pellicles were stained with 2 mL of crystal violet solution (CV, 1%) for 10 min. The excess staining solution was cautiously removed. Finally, the pellicle bound by the staining solution was dissolved in 5 mL of acetic acid solution (33%, *v*/*v*) and diluted 100–200 times for determination of the absorbance at OD_570_ [35]. For observation of the biofilm architecture, 4 µL of the strain N23 culture was spotted on a MSgg plate (1.5% agar). The assay was performed with three independent experiments.

### 2.4. Plant Material and Growth Conditions

The tomato seed germination experiments were performed as described and modified [36]. Briefly, each treatment used 80 tomato seeds, and all seeds were surface-sterilized by treatment with 70% (*v*/*v*) ethanol for 30 s followed by 4% sodium hypochlorite for 4 min and then rinsed three times with sterile distilled water as pretreatment. Strain N23 was grown at 37 °C in LB medium with shaking at 180 rpm for 3–4 h (OD_600_ ≈ 0.8). The sterilized tomato seeds were soaked in the culture of N23 for 16 h. Non-inoculated LB was used as negative control. After soaking, the tomato seeds were placed in a water agar plate (WA, 10 g agar per liter) and incubated in the 25 °C illumination incubator. Respectively, these seeds were soaked with ddH_2_O and the LB broth as controls. Three days later, the rate of germination was calculated based on the formula: germination rate (%) = (the number of germinated seed/the total number of the seed) × 100%. Each treatment was set up in three distinct WA plates for replications.

### 2.5. Antagonism Testing of B. velezensis N23 in Planta

The antagonism testing was performed as described and modified [37]. The pathogenic fungus *Colletotrichum* sp. was cultured on a PDA plate at 27 °C for ten days. The bacterial suspensions of *B. velezensis* N23 were prepared as described above. The fruits were bought from the nearby supermarket. Briefly, healthy fruits were surface-sterilized with 75% alcohol for one minute and washed twice with distilled water. The control and treated fruits were soaked in sterilized water and bacterial fermentation of N23 for five minutes, respectively. Then, the fruits were air-dried on a benchtop. For antagonism tests in planta, a uniform wound was made at the fruit using a sterile nail. We then inoculated a 5 mm-diameter mycelium block or placed 5 μL of spore suspension (1 × 10^6^ spores/mL) of pathogenic fungi *Colletotrichum* sp. on the fruit wound. The fruits treated were incubated in an incubator with 25 °C, 98% humidity, a 12 h light/12 h dark for 7–10 days. Five fruits were selected for each treatment and the experiment was carried out three times independently. The areas of the lesion were measured and evaluated for disease prevention effects based on the lesion areas seven days later. The lesion areas were calculated based on the following formula: lesion area = L × W. L: the length of a lesion; W: the width of a lesion. The assay was performed with three independent experiments.

### 2.6. Genome Sequencing, Assembly and Annotation of B. velezensis N23

For DNA extraction, a single colony of N23 was incubated and cultured in LB liquid medium at 37 °C overnight. Genomic DNA was extracted using the modified SDS lysis method. The concentration and integrity were measured with a Nanodrop One (Thermo Scientific, Waltham, MA, USA) spectrophotometer [38]. Subsequently, the DNA was sequenced using Illumina and Oxford Nanopore technologies by Wuhan Benagen Technology Solutions Co., Ltd. (Wuhan, China). In order to perform Illumina sequencing, the DNA was sheared to 350 bp using a Covaris for library construction. To ensure the quality of the DNA, it was checked using the Bioanalyzer 2100 system (Agilent Technologies, Santa Clara, CA, USA). A paired-end DNA library was created using the TruSeq DNA Sample Preparation Guide for Illumina kit, and sequencing was carried out on an Illumina NovaSeq 6000 using 150 bp reads. To obtain high-quality reads, the adapter and low-quality reads were removed and filtered using SOAPnuke v2.1.2 with default parameters [39]. For the long-read sequencing, genomic DNA was purified and directly constructed into a library using a ligation sequencing kit (SQK-LSK109, Oxford Nanopore technologies, Oxford, UK). The DNA library was barcoded using the ONT standard protocol with the native barcoding expansion 1–12 kit. It was loaded onto r9.4.1 flow cells and run on a PromethION48. Raw sequence was processed using Guppy v 5.0.16 with default setting for base-calling, barcode segmentation, and adapter sequence removal. Finally, the high-quality Illumina sequencing data and long-read sequences were assembled into a complete sequence using Unicycler v.0.4.9 with the default settings [40]. The annotation step was performed as previously reported. Briefly, the Prokka v.1.11 software used to predict the protein-coding genes and functional annotation was performed using the Basic Local Alignment Search Tool (BLAST) against the NCBI nr protein database, Kyoto Encyclopedia of Genes and Genomes (KEGG) database, Cluster of Orthologous Groups of proteins (COG), and InterPro database [41].

### 2.7. Phylogenetic Identification of Strain N23 Based on 16S rDNA, gyrA Gene Sequence, and Genome Sequence

Identification of strain N23 was performed by the analysis of 16S rDNA and *gyrA* gene sequences. The 16S rDNA gene was amplified with the primers 63F (5′-CAGGCCTAACACATGCAAGTC-3′) and 1387R (5′-GGGCGGWGTGTACAAGGC-3′), which is specific for bacteria 16S rDNA. A part of the *gyrA* gene was amplified with the primers p-gyrA-f (5′-CAGTCAGGAAATGCGTACGTCCTT-3′) and p-gyrA-r (5′-CAAGGTAATGCTCCAGGCATTGCT-3′). Amplification was performed in 20 µL reactions using a A8 FastHiFi DNA Polymerase kit (Aidlab Biotechnologies Co., Ltd., Beijing, China) with the following thermocycler protocol which included an initial denaturation at 95 °C for 4 min followed by 25 cycles of denaturation at 95 °C for 30 s, annealing at 55 °C for 30 s, and extension at 72 °C for 1 min 40 s, and a final extension at 72 °C for 10 min. PCR products was sent to Sangon Biotech (Shanghai) Co., Ltd. (Shanghai, China). for sequencing. DNA sequence homology searches were performed using the online BLAST program. Multiple alignments with sequences of closely related *Bacillus* strains and sequence similarity calculations were performed using CLUSTAL W. The phylogenetic tree of strain N23 based on 16S rDNA and *gyrA* gene was constructed using the neighbor-joining method of the Mega 5.0 software package. A bootstrap analysis of 1000 replicates was carried out.

The pan-genomic analysis was performed by using the PGAP analysis pipeline. The single core-gene was extracted from the pangenome using our own script [42]. A maximum-likelihood (ML) phylogenetic tree of *Bacillus* was established based on the 851 single-copy core proteins sequences shared by *Bacillus* genomes and the genome of *Pacibacillus polymyxa* M1 based on the following methods: (1) the mafft (version 7.310) software was accepted to perform the multiple alignment of amino acid sequences [42]; (2) the Gblocks method was selected to perform the selecting the conserved blocks from multiple alignment of proteins [43]; (3) the RaxML software (version 8.2.10) used for constructing the ML tree using PROTGAMMALGX model with 100 bootstrap replicates [41]. The tree was visualized using iTOL online software (http://itol.embl.de/, accessed on 2 October 2023). The average nucleotide identity was calculated using online JspeciesWS software (https://jspecies.ribohost.com/jspeciesws, accessed on 2 October 2023) [44]. The gene clusters for antibiotic synthesis were predicted using the AntiSMASH7.01 online prediction software package with default parameters. The genome sequences of N23 were deposited in the NCBI database under the accession numbers CP137889.1. All sequences involved in the study are available from the NCBI database.

### 2.8. PCR Detection of Antibiotic Biosynthesis Genes

Genomic DNA extraction and amplification of three antibiotic biosynthesis genes were performed using specific primers, which were listed in Table S1. The PCR amplifications were carried out in a 20 µL reaction mixture. The reaction mixture consisted of 2×Taq PCR MasterMix (10 µL) (Aidlab Biotechnologies Co., Ltd., Beijing, China), high-purity sterile water (8 µL), 0.5 µL of each Forward and Reverse primer (10 nmol/µL) and 1 µL of template DNA. The applications were performed in a thermocycler (Eppendorf nexus GX2, Hamburg, Germany) using the following PCR conditions: 95 °C for 4 min, 30 cycles of 94 °C for 1 min, annealing temperature for 1 min, 72 °C extension for 1 min, and a final extension at 70 °C for 5 min. The annealing temperatures were 52 °C, 58 °C, and 52 °C for ituA-F/ituA-R, srfA-F/srfA-R, and fenA-F/fenA-R primers, respectively. The amplification products were analyzed by electrophoresis in a 1.8% (*w*/*v*) agarose gel. Subsequently, the PCR products were sent to Sangon Biotech (Shanghai) Co., Ltd. for sequencing. The sequencing results were compared using the BLAST program in the GenBank nucleotide database from the National Center for Biotechnology Information (http://www.ncbi.nlm.nih.gov/, accessed on 20 November 2023).

### 2.9. Isolation and Characterization of Antifungal Lipopeptides

The lipopeptide crude extracts were obtained according to the method described by Luo et al. [28]. The pre-culture was grown in LB medium overnight. Then, 100 μL of the culture was transferred to 50 mL of Landy medium and incubated at 28 °C and 200 rpm for approximately 72 h. The cell-free supernatant was collected by centrifugation at 6000× *g* for 10 min. Afterwards, six grams of XAD-16 adsorbent resin (Amberlite™, Sigma-Aldrich, St. Louis, MO, USA) held in a column was washed with 50 mL of deionized water to remove salts. The cell-free supernatant was loaded onto the XAD16 resin column, washed with deionized water, and eluted with 14 mL of 100% methanol (Thermo scientific). The lipopeptide crude extracts were then dried with a rotary evaporator and dissolved in 1 mL of methanol. Subsequently, the lipopeptide crude extracts were filtered through a membrane with a pore size of 0.22 μm. To explore the antifungal activity of the lipopeptide crude extracts from N23 against plant pathogens, a technique similar to dual culture analysis was employed. Ten-microliter aliquots of the lipopeptide crude extracts or methanol (control) were spread on the PDA.

### 2.10. Statistical Analysis

The assay was performed with three independent experiments. Statistical results are expressed as mean values and standard deviations. Statistical significance was carried out via Student’s *t* test in GraphPad Prism 8 (*, *p* < 0.05; **, *p* < 0.01).

## 3. Results

### 3.1. Biocontrol and Plant Growth Promoting Activity of Strain N23

**Figure 1:**
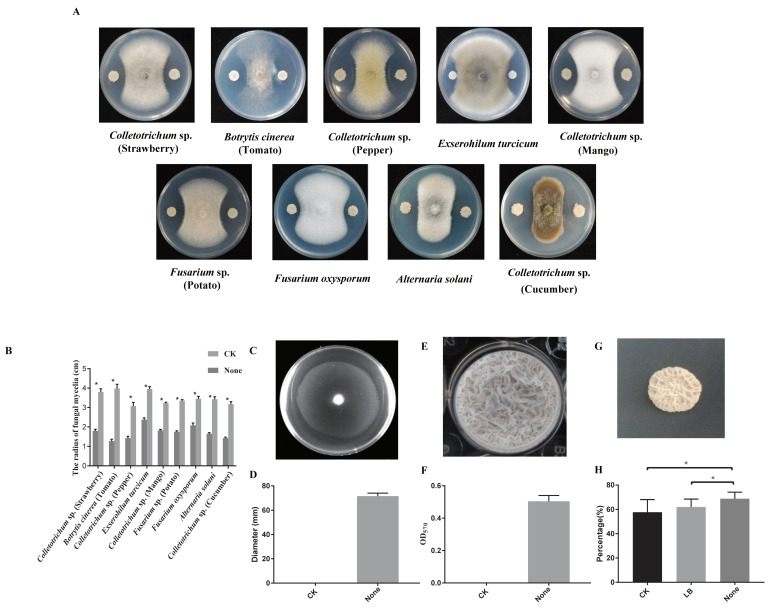
The various features of *B. velezensis* N23 (**A**) The antagonistic activity of N23 against nine pathogenic fungi on the PDA plate was assessed. (**B**) The quantification of antagonistic activity of N23 was performed. (**C**) The swarming ability of N23 was assessed. (**D**) The swarming ability of N23 on LB plates was measured five hours later. The data are expressed as the mean ± SD (*n* = 5). CK represents the LB medium and N23 represents the strain. (**E**) Pellicle formation by N23 on MSgg liquid media was observed. (**F**) Biofilm formation by N23 was quantified. The data are expressed as the mean ± SD (*n* = 6). (**G**) The colony morphology of strain N23 on MSgg plates was monitored. (**H**) The effect of N23 treatments on tomato seed germination was evaluated. CK represents the control (sterile water). LB represents the LB broth. N23 represent the biocontrol strain. Error bars indicate ± SD of three replicates. Each column indicates significant differences level of confidence according to a two-tailed *t* test. (* *p* < 0.05).

The strain N23, isolated from the contaminative plate, shows remarkable antagonistic activity against strawberry and pepper anthracnose. Additionally, strain N23 was examined for its antagonistic potential against other fungal pathogens. The results demonstrated that strain N23 significantly inhibited the mycelial growth of *Colletotrichum* sp., *Botrytis cinerea*, *Exserohilum turcicum*, *Alternaria solani*, and *Fusarium oxysporum*, as indicated by a clear inhibition zone and the average growth radius of pathogenic fungi are reduced to less than 2.5 cm (Figure 1A,B). Overall, strain N23 exhibited broad-spectrum antagonistic activities.

In the rhizosphere, *Bacillus* bacteria rely on their ability to detect and navigate towards plant compounds that are secreted in order to colonize the roots. This movement is crucial for the bacteria to find nutrient-rich areas and establish a successful interaction with the roots [45]. To gain insights into the cell motility of the strain N23, we investigated its swarming motilities. After five hours of growth, the strain N23 exhibited excellent swarming motility and colonized over half of the plate (Figure 1C,D). This suggests that the strain N23 may have a stronger ability to colonize. Bacteria often exist in the environment as cell communities known as biofilms, which are necessary for effective colonization of plant roots and protection against unfavorable conditions [46,47]. Therefore, biofilm formation is a prerequisite for effective *Bacillus* activity [48]. We also tested the biofilm formation of the strain N23. As expected, the strain N23 formed colonies with dense wrinkles, compact structures on a MSgg plate, and robust, wrinkled pellicles in MSgg liquid medium (Figure 1E–G). Over a period of 0–48 h, the level of cells accumulated in the biofilm increased approximately fivefold for the strain N23. Meanwhile, we determined the growth-promoting activity of the strain N23 by analyzing tomato seed germination. The results showed that inoculation with the bacterial strains N23 led to increased tomato seed germination compared to the control. Specifically, the strain N23 treatment significantly increased the germination rate of tomato seeds by 19.28% compared to untreated seeds. In summary, we have found a strain N23, which exhibits broad-spectrum antagonistic activities and potential plant growth-promoting properties. Above all, the strain N23 is a beneficial microbe with potential for biotechnological applications.

### 3.2. Bioassay against Colletotrichum sp. in Pepper

**Figure 2:**
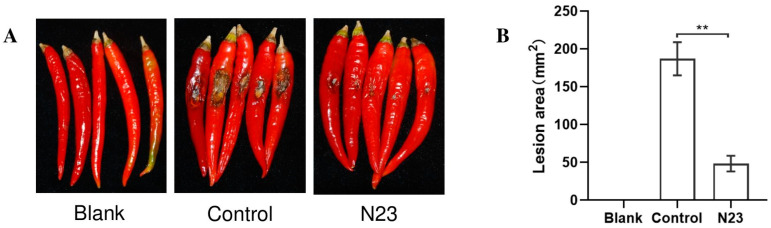
In planta biological control of anthracnose in pepper fruits using bacterium suspension of *B. velezensis* N23 (**A**) The representative pepper fruits showed the reduction in anthracnose severity in treatments when N23 were applied. (**B**) Disease severity evaluation of pepper anthracnose. Disease severity was expressed in number of lesions/mm^2^. The different number of asterisks indicates significant differences (** < 0.01) using Student’s *t*-test. The pictures were taken seven days after the inoculation of *Colletotrichum* sp.

Strain N23 is considered a potential candidate for biological control of plant pathogens based on the antifungal activities observed in the plate confrontation assay, especially for the *Colletotrichum* (Figure 1). To evaluate the in planta biological control of anthracnose caused by *Colletotrichum* sp., a bacterium suspension of N23 was used. The results displayed that the pepper was infected by the phytopathogen displayed light brown lesions (Figure 2A). The average number of lesions/mm^2^ caused by *Colletotrichum* sp. was 187.14 ± 58.21 mm^2^ in the control group, while 48.42 ± 27.33 mm^2^ in the N23-treated group (Figure 2B). The application of N23 bacterium suspension significantly reduced the number of lesions/mm^2^. And then we also obtained the similar results for controlling the strawberry anthracnose using the bacterium suspension of N23. Strawberry fruit infected by the studied phytopathogen showed typically light brown lesions (Figure S1). Meantime, the lesions/cm^2^ of *Colletotrichum* sp. was 4.81 ± 0.35 cm^2^ in control fruit and 1.35 ± 0.13 cm^2^ in N23-treated fruit. The application of N23 bacterium suspension significantly reduced the number of lesions areas (*p* < 0.01). These results clearly indicated the efficacy of N23 in reducing the severity of anthracnose caused by *Colletotrichum* and N23 showed its potential ability as a biological control agent.

### 3.3. The Genome Structure of Strain N23

The genome of *B. velevensis* N23 was sequenced to characterize and identify the genomic features. The general *B. velevensis* N23 genomic features were analyzed and summarized in Table 1 and Figure S2. The genome of strain N23 consisted of a single circular chromosome with a length of 4,014,215 bp and GC content of 46.5%; no plasmid was found. A total of 3883 protein-coding genes, which were composed of 2658 genes assigned a putative function and 1225 genes predicted to encode hypothetical proteins, were predicted in COG databases. The average length of protein-coding genes was 915 bp, and the total length of protein-coding genes accounted for 88.48% of the genomic sequence. Additionally, 27 rRNA genes and 86 tRNA-coding genes were annotated in the chromosome sequence.

### 3.4. Identification of Biocontrol Strain N23

**Figure 3:**
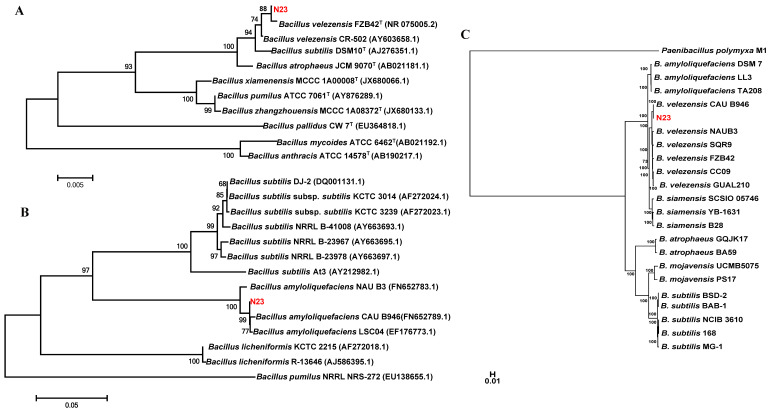
Phylogenetic analysis of strain N23 based on 16S rDNA gene sequence (**A**), *gyrA* gene sequence (**B**), and whole-genome sequence (**C**). The phylogenetic trees were constructed with the neighbor-joining method by using the software package MEGA version 5.0 after multiple sequences alignments by Clustal W. Gene bank accession numbers of each bacterial strain are shown in parentheses. The scale bar represents the number of substitutions per base position. The letter T represent type strains.

To further identify strain N23, a phylogenetic analysis was conducted using its 16S rRNA gene and *gyrA* sequences. The alignment of 16S rDNA sequences revealed a high similarity between N23 and *B. velezensis*. Additionally, the phylogenetic tree showed that the 16S rDNA sequence of strain N23 clustered with other *B. velezensis* strains (Figure 3A). A similar result was obtained from the phylogenetic analysis of a partial *gyrA* sequence, which has previously been proven effective in distinguishing closely related taxa of the *B. subtilis* group [49]. The analysis also indicated a close relation between strain N23 and *Bacillus amyloliquefaciens* group strains (Figure 3B). Furthermore, a phylogenetic tree of the genome was constructed based on the concatenation of 851 single-copy core genes present in all genomes, using the maximum likelihood (ML) method and rooted in *Paenibacillus polymyxa* M1. The results demonstrated that strain N23 and the other *B. velezensis* strains occupied the same evolutionary clade (Figure 3C). Moreover, the genome relatedness of strain N23 was assessed using average nucleotide identity (ANI) values. The findings revealed that the genomes have a high similarity between the strain N23 and other *B. velezensis* strains, with an average nucleotide identity of over 97%. Notably, strain N23 displayed a higher nucleotide identity (97.58%) with strain FZB42, which was classified as a type strain for the *B. velezensis* species (Table 2). Consequently, strain N23 was identified as *B. velezensis* (Figure 3 and Table 2).

### 3.5. Effect of B. velezensis N23 Lipopeptide Crude Extracts on Plant Pathogens

**Figure 4:**
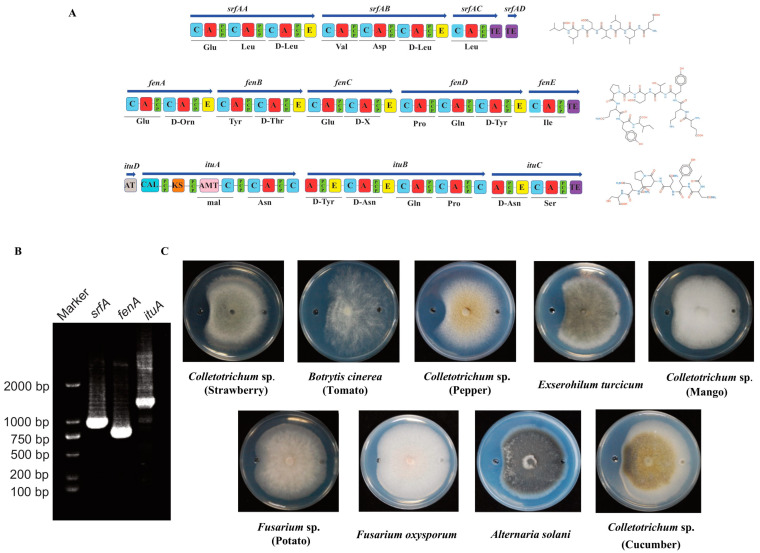
Identification of lipopeptides antibiotics produced by strain N23. (**A**) Gene clusters and predicted structure of lipopeptide including surfactin, iturin, and fengycin in the genome of N23. (**B**) PCR amplification of N23 lipopeptides genes of surfactin, fengycin, and iturin. (**C**) Antagonistic assay of the lipopeptide crude extracts of strain N23 against various pathogens. The right represents the methanol (CK) and the left represents the lipopeptide crude extracts.

*Bacillus* lipopeptides has been suggested that different lipopeptides can act synergistically to inhibit the growth of phytopathogens. Therefore, lipopeptides produced by the *Bacillus* strains attracted considerable attention [50,51]. Firstly, we searched the lipopeptides gene clusters in the genome sequences of N23. The genomic analysis showed that the genome of N23 contains three non-ribosomal gene clusters, *srf*, *fen*, and *itu*, which are responsible for the synthesis of three known lipopeptides (surfactin, fengycin, and iturin), respectively (Figure 4A). Meantime, we also used three primer pairs for PCR amplification to detect biosynthetic genes in the genomic DNA to determine the potential lipopeptide compounds produced by strain N23. Amplicons of the expected size were obtained with primer pairs designed to detect genes involved in the biosynthesis of surfactin, fengycin, and iturin (Figure 4B). The DNA sequences obtained from these amplicons confirmed the identity of these genes. Analysis of sequence from PCR product with three primer pairs showed a 100%, 100%, and 100% identity with a region of the surfactin, fengycin, and iturin biosynthesis gene cluster. Furthermore, we compared the consistency of gene clusters in the synthesis gene cluster of lipopeptide compounds surfactin, fengycin, and iturin between strain N23 and type strain *B. velezensis* FZB42. The results showed these cluster amino acid sequences had a high consistency (Table 3). We determined that lipopeptide antibiotics produced by N23 include the surfactin, iturin, and fengycin families. To test the hypothesis, the lipopeptide crude extracts of *B. velezensis* N23 were preparation according to the previous method [28]. The antagonistic assay of the lipopeptide crude extracts was conducted on the plate. As expected, the lipopeptide crude extracts showed the ability to against various pathogens (Figure 4C).

## 4. Discussion

*B. velevensis* is well-known for its biocontrol functions such as colonizing in planta, suppressing the pathogens, and inducing plant immune [12]. Although many studies showed the potential ability of *B. velevensis* against some phytopathogens, there were fewer studies on its impact on controlling *Colletotrichum* sp. causing anthracnose disease. Here, we isolated a strain N23 which had obvious inhibitory effect on *Colletotrichum* sp. in vitro and in planta, and it was identified as *B. velezensis* based on its 16S rRNA gene and *gyrA* gene sequence analysis. As a molecular marker, 16S rDNA is widely used for strain identification, but the sensitivity of this method for sub-genus distinction is markedly decreased. For example, the biocontrol strain was classified as *B. subtilis*, based on sequence homology of the 16S rRNA and metabolic profiles. However, the strains clustered with the *B. amyloliquefaciens* group based on the most reliable and accurate multilocus phylogeny analysis [52]. In the era of whole-genome sequencing, core gene sequences alignment, to identify bacteria subspecies, is more reliable. For instance, *B. amyloliquefaciens* FZB42 and *B. amyloliquefaciens* PG12 were reclassified as *B. velezensis* [41,53]. In our study, we also classified the strain N23 as *B. velezensis.*

A promising strain as a potential biocontrol agent usually has a broad-range biological activities [33,54,55]. Biocontrol bacteria exerted their biocontrol functions in the field mainly by colonizing the surface of plants, competing with pathogenic bacteria, inducing plant disease resistance, and maintaining microbiomic stability [12,20,35]. Up to now, *Bacillus* spp. have attracted most attention in studying biological control microorganisms as commercial products. *B. velezensis* has been widely regarded as a good kind of beneficial microbial preparation, based on promoting plant growth, inducing plant disease resistance, and controlling plant diseases [6,12,27,56]. In this study, dual-culture tests showed that the strain *B. velezensis* N23 has strong inhibitory effect against pathogens among the species of *Colletotrichum* sp. and *Fusarium* sp., *Botrytis cinerea*, *Exserohilum turcicum*, and *Alternaria solani* on PDA plates (Figure 1A,B). However, we discovered that the lipopeptide crude extracts of the N23 have a significant effect against *Colletotrichum* sp. and *Exserohilum turcicum* (Figure 4C). Subsequently, we found that there was a similar effect in controlling anthracnose caused by *Colletotrichum* sp. in pepper and strawberry fruit. In contrast, the lesion of pepper is more obvious than the strawberry. But the strain N23 also displayed significant inhibition to strawberry anthracnose (Figure 2 and Figure S1). These results suggested that *B. velezensis* N23 is a broad-spectrum antagonistic agent with potential applications in the control of anthracnose disease in sustainable agriculture. Certainly, field trials are needed to confirm the biocontrol ability of *B. velezensis* N23. The control efficiency of biocontrol agents in the field is also affected by many factors, such as environmental conditions and colonization ability [45,57,58]. Therefore, some factors should be considered in the application of beneficial bacteria in the field and effective measures, like exploration the conditions, scientific use of biocontrol agents, strengthening field management, should be taken to improve the biocontrol ability of biocontrol bacteria *B. velezansis* N23 for sustainable agriculture.

Lipopeptides, produced by the *B. subtilis* group, play an important role in the biocontrol of plant diseases [59,60,61]. For example, lipopeptide extracts, surfactin, and fengycin, produced by *B. subtilis* GLB191, contribute to controlling grape downy mildew by both suppressing spore germination of *Plasmopara viticola* and inducing defense gene expression of grape [20]. *B. velezensis* FZB42 is able to colonize in the lettuce rhizosphere and produce lipopeptide compounds to control the plant disease caused by the pathogen *Rhizoctonia solani* [62]. Here, we showed that the lipopeptide crude extracts of the N23 have significant protection against some pathogenic fungi such as *Colletotrichum* sp. And *B. cinerea*. Based on antiSMASH 7.0, we found the chromosome of *B. velezensis* N23 harbored many secondary metabolites including lipopeptides gene clusters such as *srfAA-AD* (responsible for the synthesis of surfactin), *fenA-E* (responsible for the synthesis of fengycin), and *ituA-D* (responsible for the synthesis of iturin) These clusters have a high sequence consistency compared to the reference strain *B. velezensis* FZB42 (Table 3). Therefore, we speculated that the strain N23 was also likely to produce these lipopeptide compounds. As expected, the lipopeptide crude extracts of the strain N23 showed antifungal activity on the PDA plate, which implied lipopeptides contributed to disease control as a kind of biocontrol factor. However, the biocontrol mechanisms and characteristics of lipopeptides crude extracts need further exploration.

## 5. Conclusions

In summary, we obtained a bacterial strain N23 from a contaminated plate which showed significant inhibition to some pathogenic fungi *Colletotrichum* sp. and *B. cinerea*. The strain N23 was classified as *B. velezensis* by using the whole genome. Based on genomic analysis and disease prevention tests on plates and fruits, lipopeptides may play a key role in the biological control of *B. velezensis* N23. *B. velezensis* N23 is a potential biocontrol agent to control pepper anthracnose. This study provided a microbial resource for agricultural application. Further comprehensive mechanism research of strain N23 needs to be designed to deepen our understanding of the biocontrol agent controlling plant disease.

## Figures and Tables

**Table 1:** Genomic features of the chromosome of *Bacillus* N23.

Attribute	*B. velezensis* N23
Genome size (bp)	4,014,251
G+C (%)	46.5
Protein-coding genes	3883
rRNA	27
tRNA	86
Protein-coding total length (bp)	3,551,679
Average length of protein-coding genes (bp)	915

**Table 2:** The average nucleotide identity (ANI) values amongst different *Bacillus* strains.

	DSM 7	LL3	TA208	CAU B946	N23	NAU-B3	SQR9	FZB42	CC09	GUAL210	SCSIO 05746	YB-1631	B28	GQJK17	BA59	UCMB5075	PS17	BSD-2	BAB-1	NCIB 3610	168	MG-1
DSM 7	100																					
LL3	99.47	100																				
TA208	99.28	99.5	100																			
CAU B946	93.39	93.26	93.27	100																		
N23	93.4	93.27	93.27	100	100																	
NAU-B3	93.4	93.28	93.31	97.52	97.52	100																
SQR9	93.09	92.99	93.02	97.18	97.18	97.82	100															
FZB42	93.37	93.3	93.3	97.58	97.58	98.26	98.39	100														
CC09	93.22	93.13	93.15	97.34	97.34	97.81	97.77	98.37	100													
GUAL210	93.34	93.27	93.3	97.6	97.6	98.14	98.08	98.42	99.07	100												
SCSIO 05746	92.99	93.01	92.85	94.1	94.1	94.1	93.9	94.15	93.69	93.96	100											
YB-1631	93.29	93.28	93.06	93.95	93.95	93.91	93.76	93.91	93.99	93.83	97.52	100										
B28	93.55	93.54	93.49	93.91	93.92	93.88	93.78	93.83	93.83	93.89	97.39	98	100									
GQJK17	76.63	76.63	76.67	76.62	76.61	76.73	76.67	76.68	76.64	76.65	76.66	76.67	76.89	100								
BA59	76.91	76.92	76.93	76.93	76.93	77	77.1	76.98	77.1	76.94	76.87	76.98	76.89	98.63	100							
UCMB5075	76.67	76.68	76.7	76.76	76.76	76.76	76.82	76.78	76.78	76.74	76.7	76.83	76.75	80.37	80.27	100						
PS17	76.12	76.18	76.18	76.13	76.13	76.3	76.74	76.23	76.29	76.24	76.21	76.21	76.23	79.81	79.81	95.39	100					
BSD-2	76.33	76.36	76.38	76.42	76.42	76.46	76.47	76.45	76.52	76.46	76.49	76.51	76.58	79.51	79.42	87.04	87.06	100				
BAB-1	76.35	76.38	76.41	76.43	76.43	76.45	76.51	76.49	76.56	76.48	76.53	76.54	76.54	79.47	79.39	87	87.08	99.97	100			
NCIB 3610	76.32	76.41	76.34	76.39	76.38	76.5	76.49	76.47	76.5	76.38	76.41	76.46	76.48	79.43	79.36	86.84	87	97.92	97.9	100		
168	76.28	76.39	76.34	76.34	76.33	76.48	76.49	76.49	76.5	76.38	76.39	76.43	76.44	79.41	79.35	86.84	86.97	97.93	97.91	100	100	
MG-1	76.11	76.26	76.17	76.21	76.21	76.28	76.35	76.28	76.28	76.22	76.31	76.32	76.38	79.32	79.26	86.66	86.82	97.78	97.76	98.33	98.33	100

**Table 3:** Comparison of the gene clusters for biosynthesis of surfactin, fengycin, and iturin between *B velezensis* N23 and *B. velezensis* FZB42.

Lipopeptide	Chromosomal Localization (from–to)	Similarity in Nucleotide Sequence (%)	Similarity in Amino Acid Sequence (%)
surfactin	323,282 nt–349,440 nt	97.7	98.0
fengycin	1,974,324 nt–2,011,993 nt	96.6	97.0
iturin	1,914,164 nt–1,951,409 nt	97.1	88.1

## Data Availability

The data presented in this study are available upon request from the corresponding author.

## Supplementary Materials

The following supporting information can be downloaded at: https://www.mdpi.com/article/10.3390/microorganisms12020294/s1, Figure S1: In vivo biological control of anthracnose in strawberry fruits using bacterium suspension of *B. velezensis* N23; Figure S2: The genome map of *B. velezensis* N23; Table S1: Specific primers for genes encoding lipopetides in *B. velezensis* N23.

## References

[1] Compant S., Duffy B., Nowak J., Clément C., Barka E.A. (2005). Use of Plant Growth-Promoting Bacteria for Biocontrol of Plant Diseases: Principles, Mechanisms of Action, and Future Prospects. Appl. Environ. Microbiol..

[2] Savary S., Laetitia W., Sarah J.P., Paul E., Neil M., Andy N. (2019). The Global Burden of Pathogens and Pests on Major Food Crops. Nat. Ecol. Evol..

[3] Wu L., Wu H.J., Qiao J.Q., Gao X.W., Rainer B. (2015). Novel Routes for Improving Biocontrol Activity of *Bacillus* Based Bioinoculants. Front. Microbiol..

[4] Yang T., Kadambot H.M.S., Liu K. (2020). Cropping Systems in Agriculture and Their Impact on Soil Health—A Review. Glob. Ecol. Conserv..

[5] He D.C., Zhan J.S., Xie L.H. (2016). Problems, Challenges and Future of Plant Disease Management: From an Ecological Point of View. J. Integr. Agric..

[6] Qiao J.Q., Wu H.J., Huo R., Gao X.W., Rainer B. (2014). Stimulation of Plant Growth and Biocontrol by *Bacillus amyloliquefaciens* subsp. plantarum Fzb42 Engineered for Improved Action. Chem. Biol. Technol. Agric..

[7] Paterson J., Ghazaleh J., Li Y., Wang Q., Samina M., Harald G., Gerard M. (2017). The Contribution of Genome Mining Strategies to the Understanding of Active Principles of PGPR Strains. FEMS Microbiol. Ecol..

[8] Prisa D., Roberto F., Damiano S. (2023). Microbial Biofertilisers in Plant Production and Resistance: A Review. Agriculture.

[9] El-Saadony M.T., Ahmed M.S., Soliman M.S., Heba M.S., Alshaymaa I.A., Mohsin M., Amira M.E., Alia A.M.E., Taia A.A.E., Shaimaa H.N. (2022). Plant Growth-Promoting Microorganisms as Biocontrol Agents of Plant Diseases: Mechanisms, Challenges and Future Perspectives. Front. Plant Sci..

[10] Borriss R. (2015). *Bacillus*, a Plant-Beneficial Bacterium. Principles of Plant-Microbe Interactions.

[11] Su Y., Liu C., Fang H., Zhang D.W. (2020). *Bacillus subtilis*: A Universal Cell Factory for Industry, Agriculture, Biomaterials and Medicine. Microb. Cell Factories.

[12] Luo L., Zhao C., Wang E., Ali R., Yin C. (2022). *Bacillus amyloliquefaciens* as an Excellent Agent for Biofertilizer and Biocontrol in Agriculture: An Overview for Its Mechanisms. Microbiol. Res..

[13] Dame Z.T., Mahfuz R., Tofazzal I. (2021). Bacilli as Sources of Agrobiotechnology: Recent Advances and Future Directions. Green Chem. Lett. Rev..

[14] Losick R., Youngman P., Piggot P.J. (1986). Genetics of Endospore Formation in *Bacillus-subtilis*. Annu. Rev. Genet..

[15] Errington J. (2003). Regulation of Endospore Formation in *Bacillus subtilis*. Nat. Rev. Microbiol..

[16] Hamdache A., Azarken R., Lamarti A., Aleu J., Collado I.G. (2013). Comparative Genome Analysis of *Bacillus* spp. and Its Relationship with Bioactive Nonribosomal Peptide Production. Phytochem. Rev..

[17] Magno-Pérez-Bryan M.C., Martínez-García P.M., Hierrezuelo J., Rodríguez-Palenzuela P., Arrebola E., Ramos C., de Vicente A., Pérez-García A., Romero D. (2015). Comparative Genomics within the Bacillus Genus Reveal the Singularities of Two Robust *Bacillus amyloliquefaciens* Biocontrol Strains. Mol. Plant-Microbe Interact..

[18] Fan H., Zhang Z., Li Y., Zhang X., Duan Y., Wang Q. (2017). Biocontrol of Bacterial Fruit Blotch by *Bacillus subtilis* 9407 Via Surfactin-Mediated Antibacterial Activity and Colonization. Front. Microbiol..

[19] Gao T.T., Ding M.Z., Yang C.H., Fan H.Y., Chai Y.R., Li Y. (2019). The Phosphotransferase System Gene Ptsh Plays an Important Role in Mnsod Production, Biofilm Formation, Swarming Motility, and Root Colonization in Bacillus Cereus 905. Res. Microbiol..

[20] Li Y., Heloir M.C., Zhang X., Geissler M., Trouvelot S., Jacquens L., Henkel M., Su X., Fang X.W., Wang Q. (2019). Surfactin and Fengycin Contribute to the Protection of a *Bacillus subtilis* Strain against Grape Downy Mildew by Both Direct Effect and Defence Stimulation. Mol. Plant Pathol..

[21] Peterson S.B., Savannah K.B., Joseph D.M. (2020). The Central Role of Interbacterial Antagonism in Bacterial Life. Curr. Biol..

[22] Rendueles O., Ghigo J.M. (2012). Multi-Species Biofilms: How to Avoid Unfriendly Neighbors. FEMS Microbiol. Rev..

[23] Chen X.H., Alexandra K., Romy S., Andreas E., Kathrin S., Isabelle H., Burkhard M., Björn V., Wolfgang R.H., Oleg R. (2007). Comparative Analysis of the Complete Genome Sequence of the Plant Growth–Promoting Bacterium *Bacillus amyloliquefaciens* Fzb42. Nat. Biotechnol..

[24] Koumoutsi A., Chen X.H., Anke H., Heiko L., Gabriele H., Peter F., Joachim V., Rainer B. (2004). Structural and Functional Characterization of Gene Clusters Directing Nonribosomal Synthesis of Bioactive Cyclic Lipopeptides in *Bacillus amyloliquefaciens* Strain FZB 42. J. Bacteriol..

[25] Romero D., de Vicente A., Rakotoaly R.H., Dufour S.E., Veening J.W., Arrebola E., Cazorla F.M., Kuipers O.P., Paquot M., Pérez-García A. (2007). The Iturin and Fengycin Families of Lipopeptides Are Key Factors in Antagonism of *Bacillus subtilis* toward *Podosphaera fusca*. Mol. Plant-Microbe Interact..

[26] Zeriouh H., Romero D., García-Gutiérrez L., Cazorla F.M., de Vicente A., Pérez-García A. (2011). The Iturin-like Lipopeptides Are Essential Components in the Biological Control Arsenal of against Bacterial Diseases of Cucurbits. Mol. Plant-Microbe Interact..

[27] Xu Z.H., Shao J.H., Li B., Yan X., Shen Q.R., Zhang R.F. (2013). Contribution of Bacillomycin D in *Bacillus amyloliquefaciens* SQR9 to Antifungal Activity and Biofilm Formation. Appl. Environ. Microbiol..

[28] Luo C.P., Zhou H.F., Zou J.C., Wang X.Y., Zhang R.S., Xiang Y.P., Chen Z.Y. (2014). Bacillomycin L and Surfactin Contribute Synergistically to the Phenotypic Features of *Bacillus subtilis* 916 and the Biocontrol of Rice Sheath Blight Induced by *Rhizoctonia solani*. Appl. Microbiol. Biotechnol..

[29] Kim Y.S., Younmi L., Wonsu C., Jungwook P., Hyeok-Tae K., Kotnala B., Jungyeon K., Yeo J.Y., Yongho J. (2021). Characterization of *Bacillus velezensis* Ak-0 as a Biocontrol Agent against Apple Bitter Rot Caused by *Colletotrichum gloeosporioides*. Sci. Rep..

[30] Bordoh P.K., Asgar A., Matthew D., Yasmeen S., Gianfranco R. (2020). A Review on the Management of Postharvest Anthracnose in Dragon Fruits Caused by *Colletotrichum* spp.. Crop Prot..

[31] Yeimmy P.R., Chiara R., Carlos D.G., Clemencia C. (2023). Green Management of Postharvest Anthracnose Caused by *Colletotrichum gloeosporioides*. J. Fungi.

[32] Branda S.S., González-Pastor J.E., Ben-Yehuda S., Losick R., Kolter R. (2001). Fruiting Body Formation by *Bacillus subtilis*. Proc. Natl. Acad. Sci. USA.

[33] Zhang X., Zhou Y.Y., Li Y., Fu X.C., Wang Q. (2017). Screening and Characterization of Endophytic *Bacillus* for Biocontrol of Grapevine Downy Mildew. Crop Prot..

[34] Chen Y., Chai Y.R., Guo J.H., Richard L. (2012). Evidence for Cyclic Di-Gmp-Mediated Signaling in *Bacillus subtilis*. J. Bacteriol..

[35] Weng J., Wang Y., Li J., Shen Q.R., Zhang R.F. (2012). Enhanced Root Colonization and Biocontrol Activity of *Bacillus amyloliquefaciens* SQR9 by Abrb Gene Disruption. Appl. Microbiol. Biotechnol..

[36] Song J., Shang L., Wang X., Xing Y., Xu W., Zhang Y., Wang T., Li H., Zhang J., Ye Z. (2021). MAPK11 Regulates Seed Germination and Aba Signaling in Tomato by Phosphorylating SNRKS. J. Exp. Bot..

[37] Than P., Jeewon P., Hyde R.K., Pongsupasamit D.S., Mongkolporn O., Taylor P.W.J. (2008). Characterization and Pathogenicity of *Colletotrichum* Species Associated with Anthracnose on Chilli (*Capsicum* spp.) in Thailand. Plant Pathol..

[38] Schallmey M., Ajay S., Owen P.W. (2004). Developments in the Use of *Bacillus* species for Industrial Production. Can. J. Microbiol..

[39] Chen Y.X., Chen Y.S., Shi C.M., Huang Z.B., Zhang Y., Li S.K., Li Y., Ye J., Yu C., Zhuo L. (2018). SOAPnuke: A Mapreduce Acceleration-Supported Software for Integrated Quality Control and Preprocessing of High-Throughput Sequencing Data. Gigascience.

[40] Phillippy A.M., Ryan R.W., Louise M.J., Claire L.G., Kathryn E.H. (2017). Unicycler: Resolving Bacterial Genome Assemblies from Short and Long Sequencing Reads. PLoS Comput. Biol..

[41] Zeng Q.C., Xie J.B., Li Y., Chen X.Y., Gu X.F., Yang P.L., Hu G.C., Wang Q. (2021). Organization, Evolution and Function of Fengycin Biosynthesis Gene Clusters in the *Bacillus amyloliquefaciens* Group. Phytopathol. Res..

[42] Katoh K., Standley D.M. (2013). Mafft Multiple Sequence Alignment Software Version 7: Improvements in Performance and Usability. Mol. Biol. Evol..

[43] Castresana J. (2000). Selection of Conserved Blocks from Multiple Alignments for Their Use in Phylogenetic Analysis. Mol. Biol. Evol..

[44] Richter M., Ramon R.M., Frank O.G., Jörg P. (2016). Jspeciesws: A Web Server for Prokaryotic Species Circumscription Based on Pairwise Genome Comparison. Bioinformatics.

[45] Rosalie A.M., Laurence T., Frédéric L., Venkatachalam L., Lucier J.F., Daniel G., Larissa C., Hera V., Harsh P.B., Pascale B.B. (2016). *Bacillus subtilis* Early Colonization of *Arabidopsis thaliana* Roots Involves Multiple Chemotaxis Receptors. Mbio.

[46] Cairns L.S., Laura H., Nicola R.S.W. (2014). Biofilm Formation by *Bacillus subtilis*: New Insights into Regulatory Strategies and Assembly Mechanisms. Mol. Microbiol..

[47] Benjamin M.S., Daniel L. (2014). Molecular Mechanisms Involved in *Bacillus subtilis* Biofilm Formation. Environ. Microbiol..

[48] Chen Y., Fang Y., Chai Y.R., Liu H.X., Roberto K., Richard L., Guo J.H. (2013). Biocontrol of Tomato Wilt Disease by *Bacillus subtilis* Isolates from Natural Environments Depends on Conserved Genes Mediating Biofilm Formation. Environ. Microbiol..

[49] Chun J., Bae K.S. (2000). Phylogenetic Analysis of *Bacillus subtilis* and Related Taxa Based on Partial Gyra Gene Sequences Gene Sequences. Antonie Van Leeuwenhoek Int. J. Gen. Mol. Microbiol..

[50] Zhao H.B., Shao D.Y., Jiang C.M., Shi J.L., Li Q., Huang Q.S., Muhammad S.R.R., Yang H., Jin M.L. (2017). Biological Activity of Lipopeptides from *Bacillus*. Appl. Microbiol. Biotechnol..

[51] Tanaka K., Yusuke A., Atsushi I., Hiromitsu N. (2015). Synergistic Effects of [Ile7]Surfactin Homologues with Bacillomycin D in Suppression of Gray Mold Disease by *Bacillus amyloliquefaciens* Biocontrol Strain Sd-32. J. Agric. Food Chem..

[52] Glaeser S.P., Peter K. (2015). Multilocus Sequence Analysis (Mlsa) in Prokaryotic Taxonomy. Syst. Appl. Microbiol..

[53] Dunlap C.A., Kim S.J., Kwon S.W., Alejandro P.R. (2016). *Bacillus velezensis* Is Not a Later Heterotypic Synonym of *Bacillus amyloliquefaciens*; *Bacillus methylotrophicus*, *Bacillus amyloliquefaciens* subsp. Plantarum and ‘*Bacillus oryzicola*’ Are Later Heterotypic Synonyms of *Bacillus Velezensis* Based on Phylogenomics. Int. J. Syst. Evol. Microbiol..

[54] Yu Y.Y., Jiang C.H., Wang C., Chen L.J., Li H.Y., Xu Q., Guo J.H. (2017). An Improved Strategy for Stable Biocontrol Agents Selecting to Control Rice Sheath Blight Caused by *Rhizoctonia solani*. Microbiol. Res..

[55] Yu Y.Y., Zhang Y.Y., Wang T., Huang T.X., Tang S.Y., Jin Y., Mi D.D., Zheng Y., Niu D.D., Guo J.H. (2023). Kurstakin Triggers Multicellular Behaviors in AR156 and Enhances Disease Control Efficacy against Rice Sheath Blight. Plant Dis..

[56] Wang H., Liu R.J., You M.P., Martin J.B., Chen Y.L. (2021). Pathogen Biocontrol Using Plant Growth-Promoting Bacteria (PGPR): Role of Bacterial Diversity. Microorganisms.

[57] Abd-Elgawad M.M.M., Tarique H.A. (2020). Factors Affecting Success of Biological Agents Used in Controlling the Plant-Parasitic Nematodes. Egypt. J. Biol. Pest Control.

[58] Velivelli S.L.S., Paul D.V.P., Kromann S.D., Barbara D.P. (2014). Biological Control Agents: From Field to Market, Problems, and Challenges. Trends Biotechnol..

[59] Ongena M., Philippe J. (2008). *Bacillus* Lipopeptides: Versatile Weapons for Plant Disease Biocontrol. Trends Microbiol..

[60] Sreedharan S.M., Niharika R., Rajni S. (2023). Microbial Lipopeptides: Properties, Mechanics and Engineering for Novel Lipopeptides. Microbiol. Res..

[61] Fazle R.M., Baek K.H. (2020). Antimicrobial Activities of Lipopeptides and Polyketides of *Bacillus velezensis* for Agricultural Applications. Molecules.

[62] Chowdhury S.P., Jenny U., Rita G., Sylvia A., Sabrina P., Kristin D., Philippe S.K., Rainer B., Anton H. (2015). Cyclic Lipopeptides of *Bacillus amyloliquefaciens* subsp. Plantarum Colonizing the Lettuce Rhizosphere Enhance Plant Defense Responses toward the Bottom Rot Pathogen *Rhizoctonia solani*. Mol. Plant-Microbe Interact..

