# Supplementary material for: Genome Sequencing and Characterization of Bacillus velezensis N23 as Biocontrol Agent against Plant Pathogens
Source: Microorganisms. 2024 Jan 30;12(2):294. doi: 10.3390/microorganisms12020294 (PMC10892835; doi:10.3390/microorganisms12020294)
Supplement: Supplementary file 1 [file microorganisms-12-00294-s001.zip › microorganisms-2830721-supplementary.pdf]

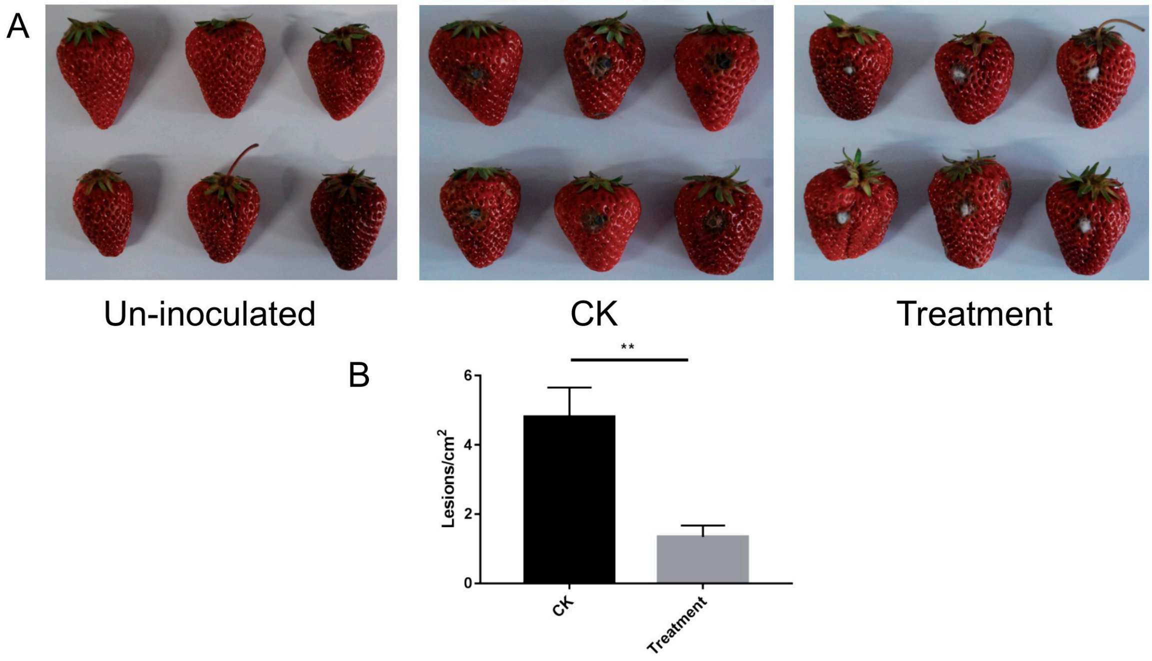


**Figure S1. In vivo biological control of anthracnose in strawberry fruits using bacterium suspension of *B. velezensis* N23.** (A) Representative fruits showing the reduction of anthracnose severity in treatments when were applied. (B) Disease severity evaluation of strawberry anthracnose. Disease severity was expressed in number of lesions/cm². Different number of asterisks indicates significant differences (** < 0.01) in t-test. Pictures were taken days after the phytopathogen inoculation.

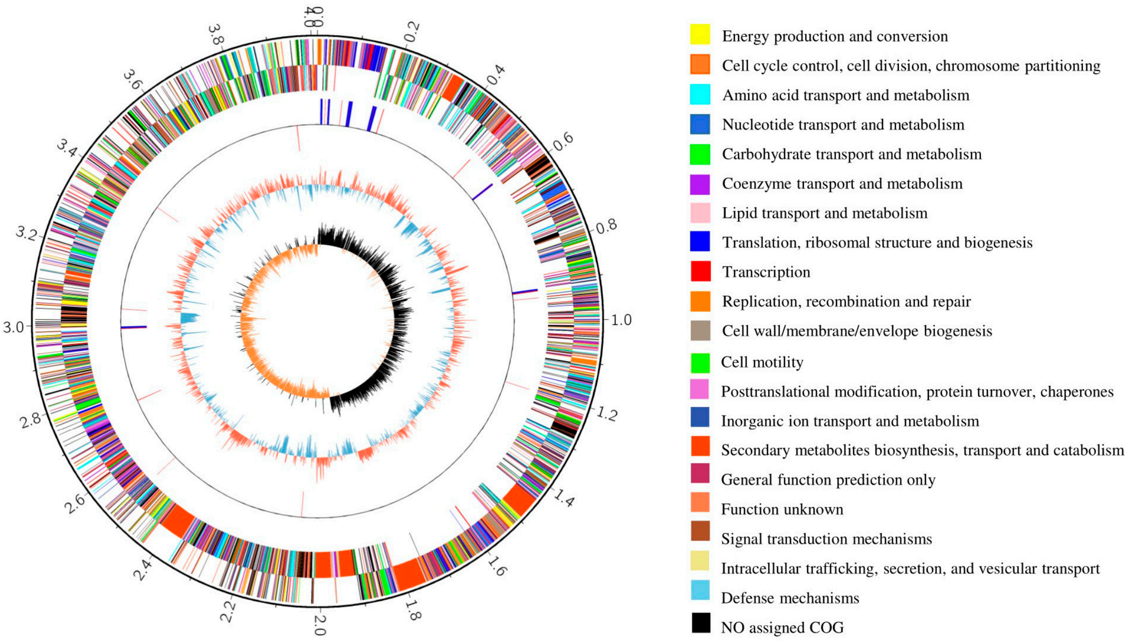


**Figure S2. The genome map of *B. velezensis* N23.** The circular diagram displays different genomic information: The first ring represents scale marks in units of Mb. The second and third rings represent protein-coding genes on the forward and reverse strands, respectively, colour-coded by functional categories. The fourth and fifth rings represent rRNA (blue) and tRNA (red) on the forward and reverse strands, respectively. The sixth ring represents GC content, with positive values shown in red and negative values shown in blue. The seventh ring represents GC skew, with above-average values shown in aquamarine and below average values shown in orange.

**Table S1 Specific primers for genes encoding lipopetides in *B. velezensis* N23.**

| Primer name | Sequence(5′-3′) |
|---|---|
| fenA-F | GTCTTGATGGTGCAGTCAGA |
| fenA-R | CTGGACCTGTTTGTCTTTGT |
| srfA-F | ACACAGATATCAGGCAAGC |
| srfA-R | GTCCCATCGTTCCTTCACA |
| ituA-F | CGGAGATCCGAAGCTGACAATAGT |
| ituA-R | GGAATTGACTGGTATACATAGGTCCCC |
